# Reducing the number and types of tobacco retail outlets in the Netherlands: Study protocol for a comprehensive mixedmethods policy evaluation

**DOI:** 10.18332/tpc/161825

**Published:** 2023-03-29

**Authors:** Gera E. Nagelhout, Nikita L. Poole, Marcel Metze, Marc C. Willemsen, Wouter Vermeulen, Floor A. van den Brand

**Affiliations:** 1 IVO Research Institute, The Hague, the Netherlands; 2 Department of Health Promotion, Care and Public Health Research Institute, Maastricht University, Maastricht, the Netherlands; 3 The Investigative Desk, Amsterdam, the Netherlands; 4 National Expertise Centre for Tobacco Control, Trimbos Institute, Utrecht, the Netherlands; 5 SEO Amsterdam Economics, Amsterdam, the Netherlands; 6 Department of Family Medicine, Care and Public Health Research Institute, Maastricht University, Maastricht, the Netherlands

**Keywords:** tobacco, policy evaluation, sales ban, social class, deprived neighborhoods, supermarkets

## Abstract

The Netherlands plans to ban tobacco sales in supermarkets in 2024. In a comprehensive policy evaluation, we aim to examine: 1) the impact of the policy on the number and types of tobacco outlets, 2) the impact on attitudes and behaviors of smoking adults and non-smoking youth, and 3) the influence of the tobacco industry on the policy process and the retail environment. In addition, our study focusses on differential effects in disadvantaged neighborhoods, where both smoking rates and tobacco outlet density are typically highest. This study brings together economic, psychological, and journalistic research methods. We investigate the impact of the new legislation on the number and type of tobacco outlets, and on the number of smokers by using routinely collected population monitoring data. We examine the impact of the legislation on smoking susceptibility of non-smoking youth and on impulse tobacco purchases by smoking adults with yearly quantitative surveys and with qualitative interviews and discussion sessions. We describe whether these impacts differ for disadvantaged versus non-disadvantaged neighborhoods. We investigate what strategies the tobacco industry uses to influence the new legislation, policy processes, and the tobacco retail environment by performing a journalistic investigation, by means of documents obtained by Freedom of Information Act (FOIA) requests, (possibly) leaked documents from insider meetings, and interviews with insiders. The methods of our evaluation can be used as a model for other comprehensive public policy evaluations.

**REGISTRATION:**

Clinical Trials ID NCT05554120, Protocol ID KWF140282021-2.

**ABBREVIATIONS:**

FOIA: Freedom of Information Act. SES-WOA: socioeconomic scores of private households. MCID: minimal clinically important difference.

## INTRODUCTION

Although smoking tobacco is highly addictive and detrimental to health^1^, tobacco is sold in many retail outlets including supermarkets, petrol stations, convenience stores, bars, and specialist tobacco shops. Most governments try to discourage the use of tobacco with, for example, warning labels on tobacco packages and high tobacco taxes, while some governments have also implemented plain packaging and tobacco display bans at the point of sale^2^. However, few countries have legislation in place to reduce the number and types of tobacco retail outlets^3,4^.

An important element of any policy to reduce the number of tobacco sale outlets is a tobacco sale licensing system. In Europe, tobacco licensing for retail outlets has been adopted in Finland, France, Hungary, Italy, and Spain, but not always with the goal of diminishing the number of tobacco outlets^3^. The introduction of licensing systems has seen a 28% reduction in the number of tobacco outlets in Finland, 31% in California, and 83% in Hungary, which has the most restrictive licensing system in Europe^3,5^. In Pennsylvania (USA), tobacco outlet density decreased by 20% three years after implementing a licensing system intended to reduce the number of outlets^6^. In New York (USA), tobacco outlet density decreased by 7% after their tobacco-free pharmacy law, especially in areas with a higher socioeconomic background^7^. New Zealand plans to decrease the number of retailers that can sell tobacco by 90% by the end of 2023^8^.

Recent meta-analyses have shown that lower levels of tobacco outlet density are associated with a lower likelihood of smoking among adolescents^9^ and adults^10^. Based on these results, we can expect that policies that reduce the number of tobacco outlets will likely diminish the visibility and availability of tobacco products, may further denormalize smoking among non-smoking youth, and thereby may contribute to the reduction of smoking in society. However, few studies have investigated the impacts of such policies on smoking behavior or prevalence. The state-monopoly licensing systems of France, Italy and Spain have not contributed to a decline in smoking prevalence^3^; but these licensing systems were introduced without the explicit goal of reducing the number of tobacco outlets and did not lead to large reductions in the number of tobacco outlets. A local ban on the sales of tobacco by pharmacies in the US did not lead to a significant reduction in smoking rates^11^, but the decision to stop the sales of tobacco by a large pharmacy chain in the US was related to a decline in household- and population-level cigarette purchasing^12^, a decline in cigarette consumption among non-daily smokers^13^, and a decline in maternal smoking during pregnancy^14^.

The Dutch government released a National Prevention Agreement in 2018^15^. One of the main goals of this Agreement is a reduction in the number of smokers to ≤5% of the adult population by the year 2040 and to create a ‘smoke-free generation’. Reducing the number of tobacco outlets is mentioned in the Agreement as one of the policy measures that the government will implement. The Netherlands does not currently have a licensing or registration system for all tobacco retailers. It is estimated that there were about 15700 tobacco retail outlets in the Netherlands in 2019, or 5.8 retail outlets per 10000 inhabitants, excluding cigarette vending machines^16^. Differences in tobacco outlet density between disadvantaged and non-disadvantaged neighborhoods have not been examined in the Netherlands, but a recent study showed that lower educated adolescents in the Netherlands are about 50% more exposed to tobacco outlets than higher educated adolescents^17^. Smoking prevalence in 2021 was 21% among Dutch adults and 17% among Dutch adolescents (aged 12–16 years), with large socioeconomic differences in smoking^18,19^.

**Figure 1:**
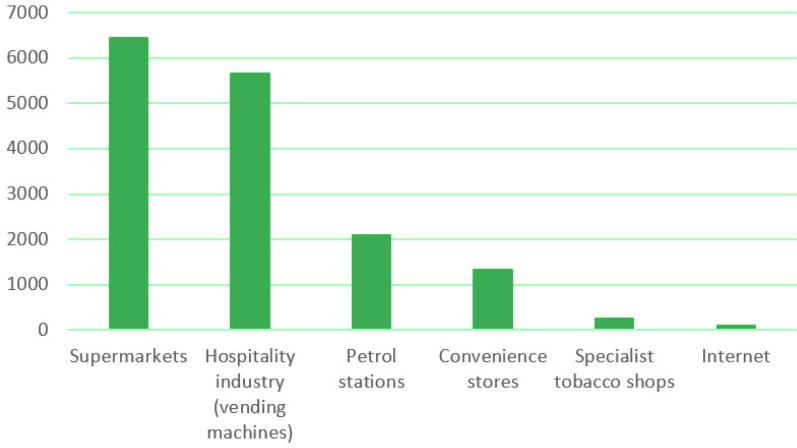
Estimation of the number of tobacco retail outlets per type in the Netherlands in 2019^17^

Since January 2022, cigarette vending machines have been banned in the Netherlands. The policy intention is to ban online sales of tobacco in July 2023, which currently account for <1% of tobacco sales in the Netherlands, and ban the sale of tobacco in supermarkets in 2024, which currently accounts for approximately 40% of tobacco outlets in the Netherlands^16^ (Figure 1). The Netherlands is the first country to ban the sale of tobacco in supermarkets. In 2030, sales of tobacco will be banned at petrol stations and in 2032 at convenience stores, so that only specialist tobacco shops can sell tobacco from 2032^20^. A study commissioned by the Dutch government found that there was support among stakeholders for limiting tobacco sales to specialist tobacco shops, but that other sellers indicated they would need time to change their business^16^. Therefore, the Dutch government decided on a stepwise approach to give convenience stores time until 2032. A follow-up study for the Dutch government predicted that the number of tobacco outlets will decrease from around 10000 before the supermarket sales ban, to 4400 in 2024, and 1440 when only specialist tobacco shops can sell tobacco^21^.

The tobacco industry is known to try to stop, delay, or weaken legislation before it is implemented, and tries to influence the tobacco retail environment to their advantage^22,23^. Both in Norway and in Scotland, a tobacco licensing system was proposed in parliament^3^. However, after strong opposition from retailers, who were encouraged to speak out against the proposed policy by the tobacco industry, both governments chose a registration system instead of a licensing system. In both countries, the main argument against a licensing system was the ‘bureaucratic burden’ for the authorities^3^; a narrative that is already present in Dutch parliamentary letters about the tobacco retail policy plans^24^. If the Dutch government indeed decides to restrict only the types of tobacco retail outlets and not set a limit on the number of outlets with tobacco licenses, the number of specialist tobacco shops may increase when the sale of tobacco is banned in supermarkets^21^. If this happens, the total reduction in tobacco retail outlets may be much less than with a licensing system.

The overall aim of our study is to evaluate the implementation of new legislation to reduce the number and types of tobacco outlets in the Netherlands, up until and including the ban on sales of tobacco in supermarkets. Our study brings together a unique combination of economic, psychological, and journalistic research methods. In a comprehensive policy evaluation, we aim to examine: 1) the impact of the policy on the number and types of tobacco outlets; 2) the impact on attitudes, smoking, and purchase behaviors of smoking adults and non-smoking youth; and 3) the influence of the tobacco industry on the policy process and the retail environment. In addition, our study focusses on differential effects in disadvantaged neighborhoods, where both smoking rates and tobacco outlet density are typically highest^25,26^.

## METHODS

**Figure 2:**
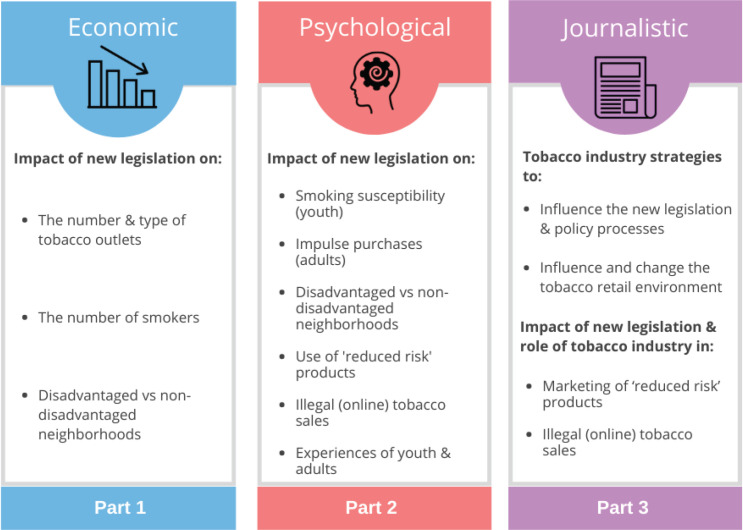
Overview of the three parts of our policy evaluation

Our study consists of three parts. See Figure 2 for an overview with the research focus per part.

### Part 1: Impact on the number and type of tobacco outlets and the number of smokers

*Research questions*

Part 1 of our study answers the following research questions:

- What is the impact of the new legislation on the number and type of tobacco outlets?
- What is the impact of the new legislation for supermarkets on the number of smokers?
- Does this impact differ for disadvantaged versus non-disadvantaged neighborhoods?

*Data*

To assess the impact of the sales ban, a time series of the number of tobacco outlets is used, distinguished by type of outlet, from Locatus. Locatus is a company which collects information about retail locations on a daily basis by a professional field service. They distinguish the following points of sale of tobacco^a^:

- Supermarkets;
- Mini supermarkets;
- Petrol stations (staffed);
- Convenience stores: they sell tobacco and convenience articles like journals, lottery tickets, office supplies, greeting cards, telephone accessories and candies;
- Tobacco specialty shops (with more than 70% of turnover from tobacco sales); and
- Night shops.

We use data from Locatus for the period 2018–2025 at the level of postal codes.

Smoking prevalence data is used at the individual level. Data at the individual level can more accurately control for differences in the characteristics of the individual. Moreover, using individual-level data enables us to evaluate heterogeneous effects between subgroups of smokers. We use existing data from the lifestyle monitor of Statistics Netherlands (15000 observations annually) and the polling station survey among students (8000 observations every four years). For each individual we link the area in which they live.

Data is used from a study which classifies neighborhoods according to the quality of living: the ‘livability measure’^27,28^. The livability measure is a monitoring instrument for quality of life at the level of 100×100 m, measuring the following sub-dimensions: physical environment, housing stock, services, social cohesion and nuisance, and insecurity. The measure consists of a resident judgement assessment model and a housing market behavioral model. The former is a residents’ assessment of how pleasant they find it to live in their neighborhood, the latter an analysis of transactions on the housing market to determine to what extent people are willing to live in a specific place. We use the classification ‘vulnerable neighborhoods’ to identify disadvantaged neighborhoods.

*Analyses*

We will describe the trends in the number of tobacco outlets from 2018 forwards, distinguished by type of retail outlet. In 2024, the number of supermarkets selling tobacco will decline to zero because of the ban on tobacco sales in supermarkets. From 2024, the number of other types of tobacco outlets might increase. In anticipation of the sales ban for supermarkets, this might even occur before 2024. Observation of the trends gives us an indication of the extent of substitution of supermarkets by other types of tobacco outlets. We also describe the development of the number of tobacco outlets in postal codes with and without supermarkets. New tobacco outlets in areas with supermarkets are likely to be replacements of supermarkets. We also investigate the development of the number of supermarkets in disadvantaged neighborhoods and non-disadvantaged neighborhoods to see whether access to supermarkets remains in all neighborhoods.

The impact of the supermarket tobacco sales ban is determined with a regression analysis. To assess the causal effect of the sales ban in supermarkets, we explain the change in the density of tobacco outlets per area by the density of supermarkets. Due to the policy, the number of supermarkets where tobacco is sold will decrease to zero in 2024. Districts with a relatively large number of supermarkets are therefore expected to experience a greater decline in the number of tobacco outlets.

We describe trends in smoking behavior in the Dutch population. These trends are described separately by type of region (more versus less densely populated or disadvantaged versus non-disadvantaged) and by individual characteristics (age, sex, socioeconomic status, heavy smoker or light smoker).

The effect of the change in the density of tobacco outlets on the number of smokers is estimated using an instrumental variable (IV) regression. This is necessary because the change in density of tobacco retail outlets is also determined by changes in the local demand for tobacco products. Hence, simply regressing changes in smoking behavior on changes in the number of tobacco retail outlets produces biased results.

The IV-technique is widely used in economics because of the difficulty of doing controlled experiments in economics^29^. The IV-technique has gained popularity in medicine, and is used for example to correct for non-compliance in randomized controlled trials^30,31^. An IV analysis offers a non-experimental alternative based on many of the same principles as a randomized controlled trial. IV analysis relies on finding a naturally varying phenomenon, related to treatment but not to the outcome, except through the effect of treatment itself, and then using this phenomenon as a proxy for the confounded treatment variable. One can then estimate how much the variation in the treatment variable that is induced by the instrument – and only that induced variation – affects the outcome measure.

Our IV-strategy relies on the change in density of tobacco retail outlets that is driven by the ban on sales of tobacco in supermarkets, which is a shock in supply that is independent from changes in demand. The density of supermarkets per area prior to the introduction of the policy is used as an instrument. If it is established in the first step that the change in tobacco outlet density is strongly related to the density of supermarkets prior to the policy change, the supermarket density can be used as an instrument. The supermarket density is then a suitable instrument as it is related to treatment (the change in the density of tobacco outlets) but is not related directly to the outcome measure (the change in smoking behavior).

Causal effects are estimated in two steps. In the first step, we predict the development of tobacco outlet density in each area by the number of supermarkets in 2018 in that area and the nationwide growth in the area. In the second step, we use this predicted number as an explanatory variable. As this predicted number is independent of the number of smokers in the area, our estimate can be interpreted as a causal effect of the change in the density of tobacco outlets.

### Part 2: Impact on attitudes and behaviors of smoking adults and non-smoking youth

*Research questions*

Part 2 of our study answers the following primary research questions:

- What is the impact of the new legislation on smoking susceptibility of non-smoking youth?
- What is the impact of the new legislation on impulse tobacco purchases by smoking adults?
- Does this impact differ for smoking adults living in disadvantaged versus non-disadvantaged neighborhoods?

Secondary research questions are:

- How do non-smoking youth and smoking adults experience the new legislation?
- To what extent does the new legislation lead to more illegal (online) sales of tobacco?
- What is the impact of the new legislation on use of ‘reduced risk’ products such as electronic cigarettes and heated tobacco?

*Data*

Our aim is to conduct yearly quantitative surveys in the autumn of 2022, 2023, 2024, and 2025. Respondents are recruited from NIPObase, a large probability-based access database that mirrors the Dutch population. NIPObase includes over 103000 respondents who regularly participate in surveys from Kantar. With NIPObase we are able to draw samples that are representative of the Dutch population according to known characteristics such as gender, age, geographical region, and household size. Respondents receive points for questions they answer, which can be exchanged for a gift certificate for themselves or a donation to a charity of choice. This procedure results in high response rates and high-quality data. Surveys are completed online. Once a respondent is included in the sample, every effort is made to track and recontact them at subsequent waves. Respondents who cannot be recontacted at a subsequent wave are replenished with a new sample of respondents, using the same inclusion criteria. These replenishment samples compensate for both the decrease in sample size, aging of the samples, and bias in demographic characteristics caused by attrition^32^. Three samples are obtained from NIPObase with quotas on gender, age, geographical region, and household size determined by information from Statistics Netherlands.

The first sample consists of 250 non-smoking youth aged 12–17 years. Youth are excluded from this survey if they have smoked (part of) a cigarette at least once a month during the last six months. For youth aged <16 years, their parents give online informed consent before the youth are asked to give online informed consent, as is according to Dutch regulations. The primary outcome measure among the sample of non-smoking youth is smoking susceptibility. This is measured with a three-item index^33^, averaging responses to: ‘Would you try smoking a cigarette if one of your best friends offered it to you?’, ‘Do you think you would smoke in the next 6 months?’, and ‘Are you curious about smoking?’. The youth questionnaire (translated from Dutch to English) can be found in the Supplementary file.

The second and third samples consist of 600 smoking adults aged ≥18 years from disadvantaged neighborhoods and 600 smoking adults aged ≥18 years from non-disadvantaged neighborhoods. Inclusion criteria are smoking at least monthly and having smoked at least 100 cigarettes in their lifetime. Respondents fill in an online informed consent before taking part in the survey. The primary outcome measure is impulse purchases of tobacco. This is measured with the question: ‘When you are shopping in a store in your neighborhood for something other than cigarettes, how often do you decide to buy cigarettes?’^34^. We distinguish between disadvantaged and non-disadvantaged neighborhoods by socioeconomic status. We use two measures as a proxy for neighborhood socioeconomic status: the ‘livability measure’^27,28^ and the socioeconomic scores of private households (SES-WOA)^35^. The SES-WOA from Statistics Netherlands is a composite measure based on the financial prosperity, the level of education and the recent employment history of households, each dimension informed by pre-existing datasets from Statistics Netherlands at the national level. Scores from both measures are available at the neighborhood level. The adult questionnaire can be found in the Supplementary file.

After the implementation of the ban in supermarkets, qualitative interviews will be carried out to provide more in-depth supplementary information to the quantitative surveys. Individual semi-structured interviews are carried out with 10 to 15 non-smoking youth aged 12–17 years, 10 to 15 smoking adults aged ≥18 years from disadvantaged neighborhoods, and 10 to 15 smoking adults aged ≥18 years from non-disadvantaged neighborhoods. Within each group variation is ensured in gender, age, education level, and cultural background. All participants receive an incentive for participation in the interview.

*Analyses*

The primary analyses will be Generalized Estimating Equations models for the youth and adult samples, separately. For the primary analyses, the second and third survey waves are compared, i.e. before and after the ban on tobacco sales in supermarkets. In secondary analyses, changes across the four survey waves are examined. With the two waves before the planned policy implementation, we can catch anticipatory actions of tobacco retailers. With the two waves after the planned policy implementation, we can still measure policy effects if policy implementation will be delayed by a year or if a transition period will be applied. If the policy is implemented as planned in 2024 (between the second and third survey wave), the fourth survey wave can be used to identify whether effects are temporary or lasting. It should be noted that with a longitudinal cohort study without a control group, we cannot be certain that changes in responses between the survey waves before the policy implementation and the survey waves after the policy implementation can be attributed to the policy change.

For the sample of non-smoking youth, the primary outcome measure is smoking susceptibility, measured on a 4-point scale. Mean scores on smoking susceptibility between survey waves 2 and 3 are compared, while adjusting for gender, age, education level, and time in sample. With an alpha of 0.05, power of 0.90, and an expected dropout of 70%, we can detect a minimal clinically important difference (MCID) of 0.25 with n=250 respondents in total.

For the sample of smoking adults, the primary outcome measure is impulse purchases of tobacco, measured on a 5-point scale. Mean scores on impulse tobacco purchases between survey waves 2 and 3 are compared and the interaction between living in a disadvantaged neighborhood or not and survey wave (change in impulse tobacco purchases) is tested, while adjusting for gender, age, education level, heaviness of smoking, living in a disadvantaged neighborhood or not, and time in sample. With an alpha of 0.025, power of 0.90, and an expected dropout of 70%, an MCID of 0.25 can be detected with n=600 respondents from disadvantaged neighborhoods and 600 from non-disadvantaged neighborhoods.

Qualitative interviews are analyzed thematically using the Framework Method^36^. Analyses will start during the interviewing phase, and new participants are interviewed until we have 15 interviews per group or until saturation is reached. Participants can choose to participate only in the interviews or to stay involved throughout the remainder of our study. Participants who want to stay involved will first meet in a discussion session with 5 to 10 participants per session. In these sessions, we discuss the results of the interviews and ask for feedback about our interpretations. This is a form of ‘member checking’ that is often useful to better understand and interpret qualitative findings. Transcripts of the discussion sessions are added to the thematic analyses of the interviews.

### Part 3: Influence of the tobacco industry on the policy process and retail environment

*Research questions*

Part 3 of our study answers the following primary research questions:

- What strategies does the tobacco industry use to influence the new legislation and policy processes?
- What strategies does the tobacco industry use to influence the tobacco retail environment?

Secondary research questions are:

- To what extent does the new legislation lead to more illegal (online) sales of tobacco and what is the role of the tobacco industry in this?
- What is the impact of the new legislation on marketing of ‘reduced risk’ products such as electronic cigarettes and heated tobacco?

*Data*

A journalistic investigation is performed of the tobacco industry’s responses to government policies concerning tobacco outlets and the retail environment. We investigate the interrelatedness of these responses with reactions to other tobacco control policies and the industry’s successes in reducing their impact. We also look into the industry’s use of proxy lobbies and on its lobbying efforts via the European Union, the coordination of lobbying efforts with other sectors, trade associations, retailers’ unions, and employers’ organizations. In this investigation, documents obtained by FOIA-requests, (possibly) leaked documents from insider meetings, and numerous (background) interviews with insiders are used.

Financial data and marketing data about the key developments in the Dutch tobacco retail environment are collected and analyzed. OSINT (open source intelligence) is used to collect financial data and marketing data. This includes annual reports, chamber of commerce data, and market reports. We collect information about industry strategies from annual reports, shareholders’ meetings, and meetings of CFOs with financial analysts (recordings of which are often publicly available). Sometimes we may acquire leaked internal strategy documents and other information may come from background interviews with former staff. This will be complemented with observations ‘on the ground’ by regularly (at least quarterly) visiting tobacco outlets, photographing new marketing features and talking to the people behind the counters.

Observations are done on the Internet, for mapping the developments in (online) sales, possibly also via ‘scraping’^37^. This technique involves systematically gathering and arranging data from a large number of websites and provides insight into the development of the number of (online) sales sites, of product pricing, of changes in product availability and of the overall ‘traffic’ (visitor number) of these websites, indicating (legal or illegal) shopping activities. The scraping software that we use was custom-made by the Dutch firm Trollrensics.

Social media campaigns are analyzed and how these influence the political debate. We investigate whether, and if so, to what extent, grassroots movements are infiltrated by industry and how (possibly fake) bottom-up campaigns are covertly started by commercial actors, such as manufacturers themselves or their affiliated PR-firms. An initial probing search using our custom-made scraping software has already led to some ‘red flags’ for orchestrated/commercially steered social media campaigns focused on EU policy documents on measures regarding ‘reduced risk’ products.

Tobacco industry activity cannot be viewed in isolation but needs to be put in a wider context. Our already running large-scale investigative research program offers such a context. In this large-scale research program, we investigate the implementation and consequences of the main policies of the National Prevention Agreement, including those concerning tobacco outlets but also those related to excise duties, plain packaging, and the display ban. In addition, major investigations into the tobacco industry’s involvement in the development of new versions of the main European tobacco directives (Tobacco Product Directive and Tobacco Tax Directive) are set up. These policy measures will have a direct impact on the tobacco retail environment, since prices, packaging and advertisement regulations affect the sales and therefore profitability of tobacco. This impacts the entire retail chain, from industry to shopkeeper.

It is also important to address the fact that in the last decade, the tobacco industry has changed its marketing strategies and is moving away from ‘burned’ tobacco to e-cigarettes and heated tobacco products, using the ‘harm reduction’ concept as a new slogan^38^. The industry has begun to roll out this strategy in western countries and may gradually introduce it in non-western parts of the world as well. For our comprehensive evaluation, a wide definition of ‘tobacco outlets’ is adopted and includes the so-called ‘reduced risk’ products.

*Analyses*

For part 3 of our study, an investigative journalism approach is used to analyze our data and answer our research questions. We have developed a structured, process-oriented investigative journalism approach over the past ten years by integrating existing journalistic techniques, recently developed datajournalistic techniques, and elements from qualitative (social science) research methodology. This has resulted in a step-by-step, structured but iterative approach which involves orientation research, the development of research plans based on central research questions, periodic internal research updates, reappraisals (if necessary) of research questions, and structured, analysis-based reporting.

## DISCUSSION

In the next four years, we will conduct this comprehensive mixed-methods policy evaluation in which we evaluate the implementation of new legislation to reduce the number and types of tobacco outlets in the Netherlands. We believe that the methods of our evaluation described above can be used as a model for other comprehensive public policy evaluations. Major strengths of our approach are the combination of economic, psychological, and journalistic research methods, the combination of quantitative and qualitative data collection and analyses, the combination of a population perspective and a focus on disadvantaged neighborhoods, and examining both smoking adults and non-smoking youth.

A challenge that we foresee in this evaluation is the integration of all evidence into a coherent narrative about our core findings with resulting policy recommendations for the Netherlands and other countries. We will start the integration process by creating an overview of complementary and contradicting findings from the three parts of our study. In the last project year, we will organize meetings with the project team, interested study participants from the qualitative discussion sessions, and relevant national experts and stakeholders. During these meetings, the attendees discuss study results and work towards formulating policy recommendations based on these results. These meetings are expected to help considerably with the integration process.

Another possible challenge is that many elements of the new tobacco outlets policy are still unknown, including the exact timing of policy implementation. A study design with multiple measurements before and after the planned policy implementation is chosen to take this into account. With the multiple measurements before the planned policy implementation, anticipatory actions of tobacco retailers can be caught. With the two measurements after the planned policy implementation, policy effects can still be measured if policy implementation will be delayed by a year or if a transition period will be applied. If the worst-case scenario happens and no policy is implemented at all, the investigative journalism part of our research will uncover how this happened, which can lead to important recommendations on how to prevent significant policy delay or policy failure in other countries and in the Netherlands in the future.

The four research organizations that collaborate in this comprehensive policy evaluation have already worked and published together in several previous research projects^16,21,39-42^. This makes us confident about a successful collaboration in the current evaluation that will likely lead to a high scientific and journalistic output.

## CONCLUSIONS

We aim to publish at least four scientific articles in open-access international peer-reviewed journals, multiple journalistic publications per year, and short accessible research updates in the form of news flashes, factsheets, and blogs. During the project, we will present our work at national and international scientific conferences. We will close our project with a final webinar in which we present the core findings of our evaluation and the resulting policy recommendations to an international audience. With these dissemination activities, we aim to inform the general public, national health funds, policy makers and scientists about the progress and findings of our comprehensive evaluation.

## Data Availability

Data sharing is not applicable to this article as no new data were created.

## Footnotes

a The data measure the number of tobacco retail outlets with some error. There is no guarantee that every retail location in each category sells tobacco products, nor can we entirely rule out tobacco sales in other types of outlets. This measurement error may bias results. Our IV-strategy, to be discussed in the next section, will be robust to measurement error.
