# Supplementary material for: Reducing the number and types of tobacco retail outlets in the Netherlands: Study protocol for a comprehensive mixedmethods policy evaluation
Source: Tob Prev Cessat. 2023 Mar 29;9:08. doi: 10.18332/tpc/161825 (PMC10054766; doi:10.18332/tpc/161825)
Supplement: Supplementary file 1 [file TPC-9-08-s1.pdf]

Supplement 1: Youth questionnaire.

Concept and instructions	Item in English
Eligibility - monthly smoking	*This is an essential question that will help to determine your eligibility. If you can't or don't wish to answer this question, you will not be able to continue.*<br>*Note: with smoking we mean: sigarettes and shag, with smoking we do not mean: cigars, e-cigarettes or waterpipe.*<br><br>Have you smoked (part of) a cigarette at least once a month in the past six months?<br>1 Yes → survey ends<br>2 No
Ever smoked	Have you ever smoked a cigarette, even just a few puffs ?<br>1 Yes<br>2 No<br>3 Don't know/Don't want to say
Smoking initiation	At what age did you smoke for the first time, including a few puffs?<br>Drop down menu 1-17<br>Don't know/Don't want to say
Frequency of smoking (cigarettes)<br>[Wave 2 onwards]	How often do you currently smoke cigarettes, including both factory-made and roll-your-own cigarettes?<br>1 Daily<br>2 Weekly<br>3 Monthly<br>4 Less than monthly, but occasionally<br>5 Not at all; I've quit smoking completely<br>9 Don't know/Don't want to say
Days per week smoking<br>[if smoking]<br>[Wave 2 onwards]	On how many days do you smoke per week?<br>Drop down menu 1-7<br>Don't know/Don't want to say
Days per month smoking<br>[if smoking]<br>[Wave 2 onwards]	On how many days do you smoke per month?<br>Drop down menu 1-31<br>Don't know/Don't want to say
Daily average number of cigarettes<br>[if smoking]<br>[Wave 2 onwards]	On the days that you smoke, how many sigarettes/shag do you smoke on average per day?<br>Drop down menu 1-100<br>Possible answers 1+.<br>Don't know/Don't want to say
Stopintentie<br><br>[if smoking]<br>[Wave 2 onwards]	And are you planning to quit smoking within the next 6 months?<br>1 Very likely<br>2 Likely<br>3 Possibly<br>4 Unlikely<br>5 Very unlikely<br>9 Don't know/Don't want to say
Quit attempts<br><br>[if smoking]<br>[Wave 2 onwards]	A serious quit attempt is one that lasts at least 24 hours.<br>How many SERIOUS quit attempts have you ever made?<br>99 Don't know/Don't want to say<br>(Range 0-98)
Acquisition of products<br><br>[if smoking]<br>[Wave 2 onwards]	During the past 30 days, how did you get your own cigarettes?<br>(Multiple answer options possible)<br>1 I bought it myself<br>2 I had someone else buy it for me<br>3 I borrowed it from someone<br>4 Someone gave it to me without my asking<br>5 Other<br>9 Don't know/Don't want to say
Location of purchase<br>[if smoking]<br>[Wave 2 onwards]	At which store or point of sale do you usually buy it?<br>[multiple answers possible]<br>1 Supermarket<br>2 Via the Internet<br>3 Bookstore(for example Primera, AKO or Bruna)<br>4 Tobacconist<br>5 Gas station<br>6 Somewhere else<br>9 Don't know/Don't want to say
Travel time to location<br>[if smoking]<br>[Wave 2 onwards]	How many minutes on average does it take to get to there?<br>[for each point-of-sale given in the previous question]<br>1 Less than 5 minutes<br>2 5-10 minutes

	3 11-20 minutes<br>4 20-30 minutes<br>5 More than 30 minutes<br>99 Don't know/Don't want to say
Perceived success if attempting to purchase cigarettes	If you, or someone your age, tried to buy cigarettes in a store, do you think you would be successful?<br>1 Yes<br>2 No<br>99 Don't know/Don't want to say
	If you, or someone your age, tried to buy cigarettes online, do you think you would be successful?<br>1 Yes<br>2 No<br>99 Don't know/Don't want to say
Susceptibility to smoking	Would you try smoking a cigarette or shag (e.g. Marlboro, Camale, Van Nelle etc.) if one of your best friends offered it to you?<br>1 Definitely Not<br>2 Probably Not<br>3 Probably Yes<br>4 Definitely Yes<br>5 Don't know/Don't want to say
	Do you think you would smoke cigarettes or shag in the next 6 months?<br>1 Definitely Not<br>2 Probably Not<br>3 Probably Yes<br>4 Definitely Yes<br>5 Don't know/Don't want to say
	Are you curious about trying cigarettes?<br>1 Definitely Not<br>2 Probably Not<br>3 Probably Yes<br>4 Definitely Yes<br>5 Don't know/Don't want to say
Attitude about smoking	What is your general opinion about smoking?<br>1 Very positive<br>2 Positive<br>3 Not positive, but not negative either<br>4 Negative<br>5 Very negative<br>9 Don't know/Don't want to say
Exposure to tobacco promotion	In the last 6 months, how often have you noticed any of the following types of tobacco promotion?<br>• Special price offers in stores<br>• Free samples<br>• Internet sites/emails/leaflets promoting cigarettes or tobacco products<br>• Signs/posters/branded items (with logo)<br>• Sponsoring by tobacco or cigarette brands (for example Marlboro, Camel, Van Nelle etc.)<br>1 Very often<br>2 Often<br>3 Sometimes<br>4 Once or twice<br>5 Never<br>9 Don't know/Don't want to say
Proportion of friends who smoke	Of the five closest friends that you spend time with on a regular basis, how many of them are smokers?<br>Drop down menu 0-5<br>Don't know/Don't want to say
Proportion overestimating the prevalence of children their age who smoke	Out of 100 children your age, how many do you think smoke cigarettes at least once a week?<br>Drop down menu 0-100<br>Don't know/Don't want to say
Parents smoking status	Do any of your parents, step-parentens or guardians smoke?<br>Here we mean smoking of all kinds of tobacco products but <u>not</u> the use of e-cigarettes of products that heat tobacco (heated tobacco procuts, heat-not-burn, IQOS).<br>1 Yes<br>2 No<br>9 Don't know/Don't want to say
Siblings smoking status	Do any of your brother or sisters smoke?<br>1 Yes

	2 No<br>3 I don't have any brothers or sisters<br>9 Don't know/Don't want to say
Approval of smoking - friends	To what extent do you agree with the statement 'Your friends disapprove of smoking'?<br>1 Strongly agree<br>2 Agree<br>3 Neither agree nor disagree<br>4 Disagree<br>5 Strongly disagree<br>9 Don't know/Don't want to say
Approval of smoking - parents	To what extent do you agree with the statement 'Your family disapproves of smoking'<br>1 Strongly agree<br>2 Agree<br>3 Neither agree nor disagree<br>4 Disagree<br>5 Strongly disagree<br>9 Don't know/Don't want to say
Exposure to second-hand smoke in the home	How often are you exposed to the tobacco smoke of others inside the home?<br>1 Never or barely ever<br>2 Less than one time a week<br>3 At least one time a week, but not every day<br>4 Every day for less than one hour per day<br>5 Every day for more than one hour per day<br>5 Don't know/Don't want to say
Exposure to second-hand smoke outside of the home	How often are you exposed to the tobacco smoke of others in places other than in your home?<br>1 Never or barely ever<br>2 Less than one time a week<br>3 At least one time a week, but not every day<br>4 Every day for less than one hour per day<br>5 Every day for more than one hour per day<br>5 Don't know/Don't want to say
Frequency of visits to points-of-sale<br>[Matrix question]	How often do you visit the following points-of-sale?<br>• Supermarket<br>• Bar or restaurant<br>• Duty-free shop (for exmaple at the airport)<br>• Bookstore (for example Primera, AKO or Bruna)<br>• Gas station<br>1 Daily<br>2 Weekly<br>3 Monthly<br>4 Less than monthly<br>5 Never<br>9 Don't know/Don't want to say
Seeing tobacco in the retail environment<br>[Matrix question]	How often have you seen the following when visiting a store in the last month:<br>• Cigarette packages at the counter or checkout<br>• Smoking accessories (for example lighters, filters, rolling paper)<br>• Advertisements for tobacco<br>• People smoking at the entrance of the store<br>• A friend buying cigarettes<br>• A family member buying cigarettes<br>1 Very often<br>2 Often<br>3 Sometimes<br>4 Rarely<br>5 Never<br>9 Don't know/Don't want to say
Ever use of e-cigarettes/HTPs	Now there follows a few questions about other products you can smoke or use.<br>E-cigarettes are tobacco0free products that evaporate liquid. This can be a liquid with or without nicotine. They are also known as electronic cigarettes.<br>Electronic products that heat tobacco instead of burning it use batteries to heat capsules, sleeves, or sticks that look like cigarettes. These are products like IQOS.<br>Have you ever used these products?<br>• E-cigarette<br>• Heated tobacco product<br>1 Yes<br>2 No<br>3 Don't know/Don't want to say<br>4 I have never heard of it before

Current use of e-cigarettes/HTPs	On how many days of the last 30 days have you used an electronic cigarette, or e-cigarette with nicotine? Other names for this are e-smoker or shisha-pen. By this we do not mean an apparatus that heats tobacco (heated tobacco product, HTP, heat-not-burn), such as IQOS.<br>Drop down menu 0-30<br>I have never heard of e-cigarettes<br>Don't know/Don't want to say
	On how many days of the last 30 days have you used an electronic cigarette, or e-cigarette without nicotine?<br>Drop down menu 0-30<br>I have never heard of e-cigarettes<br>Don't know/Don't want to say
	On how many days in the last 30 days have you used an apparatus that heats up tobacco (heated tobacco product, HTP, heat-not-burn), such as the IQOS?<br>Drop down menu 0-30<br>I have never heard of e-cigarettes<br>Don't know/Don't want to say
Acquisition of e-cigarette/HTP	During the past 30 days, how did you get your own [e-cigarette/HTP]?<br>1 I bought it myself<br>2 I had someone else buy it for me<br>3 I borrowed it from someone<br>4 Someone gave it to me without my asking<br>5 Other<br>9 Don't know/Don't want to say
Location of purchase e-cigarette/HTP	At which store or point of sale did you buy it (most often)?<br>[multiple answers possible]<br>1 Supermarket<br>2 Bar or restaurant<br>3 Duty free shopping<br>4 Via the Internet<br>5 Bookstore (for example Primera, AKO or Bruna)<br>6 Tobacconist<br>7 Vape shop<br>8 Gas station<br>9 From someone else – not at a shop or other regular point of sale<br>99 Don't know/Don't want to say
Use of alternative products and substances<br><br>[Matrix question]	Have you used any of the following in the last month?<br>• Alcohol<br>• Laughing gas<br>• Cannabis (hash, weed)<br>• Snus (a bag of tobacco ground into a moist powder placed between the cheek or lip and gums. We don't mean nicotine bags that don't contain tobacco)<br>• Nicotine pouches (sachets with a powder, nicotine in different quantities and an added taste, but without tobacco)<br>1 Yes<br>2 No<br>9 Don't know/Don't want to say
Social denormalisation	Please indicate to what extent you agree with the following statements:<br>People who are important to me believe I should not smoke.<br>1 Strongly agree<br>2 Agree<br>3 Neither agree or disagree<br>4 Disagree<br>5 Strongly disagree<br>9 Don't know/Don't want to say
	Society disapproves of smoking.<br>1 Strongly agree<br>2 Agree<br>3 Neither agree or disagree<br>4 Disagree<br>5 Strongly disagree<br>9 Don't know/Don't want to say
	There are fewer and fewer places I [would] feel comfortable smoking.<br>1 Strongly agree<br>2 Agree<br>3 Neither agree or disagree<br>4 Disagree<br>5 Strongly disagree<br>9 Don't know/Don't want to say

Support for supermarket ban	To what extent do you agree with the following statement? The sale of tobacco products in supermarkets should be completely banned.<br>1 Strongly agree<br>2 Somewhat agree<br>3 Neither agree nor disagree<br>4 Somewhat disagree<br>5 Strongly disagree<br>9 Don't know/Don't want to say
Seen or heard about smoking cessation campaign(s)	In the last 12 months about how often, if at all, have you seen or heard a smoking cessation campaign about smoking, such as the Smoke-free Generation?<br>1 Never<br>2 Rarely<br>3 Sometimes<br>4 Often<br>5 Very often<br>9 Don't know/Don't want to say
Seen or heard about smoking policy	In the last 12 months about how often, if at all, have you seen or heard of smoking policy in the news (including new on social media)?<br>1 Never<br>2 Rarely<br>3 Sometimes<br>4 Often<br>5 Very often<br>9 Don't know/Don't want to say
Thinking about smoking cessation<br>[Matrix question]<br>[if smoking]<br>[Wave 2 onwards]	To what extent have any of the following made you think about quitting smoking in the last 12 months?<br>[ask per point]<br>• A prevention message or campaign<br>• The price of cigarettes<br>• The ban on sale of tobacco products in supermarkets<br>• Smoking bans in new or more places<br>• Availability of telephone helpline/ Quitline/ information line<br>1 A great deal<br>2 A fair amount<br>3 Just a little<br>4 Not at all<br>9 Don't know/Don't want to say
Highest followed level of education – adolescents	What is the highest level of education you have followed?<br>1 None (Elementary school not completed)<br>2 Elementary school<br>3 Preparatory vocational secondary education – VMBO (bv. LTS, Kader, BB, ambachtsschool)<br>4 Preparatory vocational secondary education – VMBO-TL (bv. MAVO, MULO)<br>5 Senior secondary vocational education - MBO (bv. MEAO, MTS)<br>6 Senior secondary general education and pre-university education (bv. HAVO, VWO, Gymnasium)<br>7 Higher professional education - HBO (bv. HTS, HBO-V, PABO)<br>8 Academic higher education, Bachelor, Master (Universiteit)<br>9 Don't know/Don't want to say
Age in years<br><br>[Received from Kantar panel data]	Age in years
Sex<br><br>[Received from Kantar panel data]	Sex<br>1 Man<br>2 Woman
Highest completed level of education – parents<br><br>[Received from Kantar panel data, parent asked]	What is your highest completed level of education?
Household income<br><br>[Received from Kantar panel data]	What is your household total gross income?
Closing - Information about smoking	We would like to invite you to participate again next year with the follow-up survey.<br><br>If you want to find out more about smoking and the tobacco industry, more information is available at https://www.rokeninfo.nl/ and https://jongvolwassenen.ikstopnu.nl/

Supplement 2: Adult questionnaire.

Concept and instructions	Item in English
Eligibility – More than 100 cigarettes smoking in lifetime	*This is an essential question that will help to determine your eligibility. If you can't or don't wish to answer this question, you will not be able to continue.*<br>*Note: with smoking we mean cigarettes and shag. With smoking we do <u>not</u> mean cigars, e-cigarettes or waterpipe.*<br><br>Have you smoked a combined total of 100 or more factorymade or roll-your-own cigarettes over your lifetime?<br>1 Yes<br>2 No → survey ends<br>9 Don't know/Don't want to say → survey ends
Eligibility – monthly smoking	Do you smoke either manufactured or roll-your-own cigarettes at least monthly?<br>1 Yes<br>2 No → survey ends
Frequency smoking (cigarettes)	How often do you currently smoke cigarettes, including both factory-made and roll-your-own cigarettes?<br>1 Daily<br>2 Weekly<br>3 Monthly<br>4 Less than monthly, but occasionally [Wave 2 onwards]<br>5 Not at all; I've quit smoking completely [Wave 2 onwards]<br>9 Don't know/Don't want to say
Days per week smoking	On how many days do you smoke per week?<br>Drop down menu 1-7<br>Don't know/Don't want to say
Days per month smoking	On how many days do you smoke per month?<br>Drop down menu 1-31<br>Don't know/Don't want to say
Daily average number of cigarettes	On the days that you smoke, how many manufactured or roll-your-own cigarettes do you smoke on average per day?<br>Drop down menu 1-100<br>Don't know/Don't want to say
Nicotine dependence (HSI)	How soon after waking do you usually have your first cigarette?<br>1 5 min or less<br>2 6-30 min<br>3 31-60 min<br>4 More than 60 min<br>9 Don't know/Don't want to say<br>[with daily cigarette consumption: Heaviness of Smoking Index (HSI) from 0 to 6]
Quit attempts - ever	Have you ever tried to quit smoking?<br>1 Yes<br>2 No<br>9 Don't know/Don't want to say
Serious quit attempts	A serious quit attempt is one that lasts at least 24 hours.<br>How many SERIOUS quit attempts have you ever made?<br>Drop down menu 1-100<br>Don't know/Don't want to say
How recent last quit attempt	How long ago did your most recent quit attempt start?<br>1 Less than 1 week ago<br>2 1-4 weeks ago<br>3 1-3 months ago<br>4 4-6 months ago<br>5 7-12 months ago<br>9 Don't know/Don't want to say
Duration most recent quit attempt	How long were you quit for on your most recent quit attempt?<br>01 Less than 1 day<br>02 1-6 days<br>03 1-2 weeks<br>04 3-4 weeks<br>05 1-3 months<br>06 4-6 months<br>07 7-12 months<br>99 Don't know/Don't want to say
Intention to quit	And are you planning to quit smoking cigarettes within the next 6 months? That is…<br>1 Very likely<br>2 Likely<br>3 Possibly<br>4 Unlikely<br>5 Very unlikely<br>9 Don't know/Don't want to say

Frequency of tobacco purchase	How often do you typically buy cigarettes or roll-your-own tobacco?<br>1 Nearly every day<br>2 2-3 times a week<br>3 Once a week<br>4 2-3 times a month<br>5 Once a month<br>6 Less than once a month<br>7 Never<br>8 Don't know/Don't want to say
Impulse purchases	If you are in a store in your neighbourhood, how often do you get an urge to buy cigarettes?<br>1 Never<br>2 Rarely<br>3 Sometimes<br>4 Often<br>5 Always<br>9 Don't know/Don't want to say
	An impulse purchase is an unplanned purchase of cigarettes.<br>You are in a shop to buy something other than cigarettes. How often do you decide to buy cigarettes as an impulse purchase?<br>1 Never<br>2 Rarely<br>3 Sometimes<br>4 Often<br>5 Always<br>9 Don't know/Don't want to say
Reason for impulse purchase	What is usually the reason for making an impulse purchase?<br>1 Seeing tobacco procuts at the checkout or counter<br>2 Running low or running out<br>3 Avoiding running low<br>4 Seeing somone else buying cigarettes<br>5 Drinking alcohol<br>6 Experiencing cravings<br>7 Being around smokers<br>8 Loss of will power<br>9 Other<br>99 Don't know/Don't want to say
Location of purchase – most often<br><br>[matrix question]	Think about where you usually buy your cigarettes or roll-your-own tobacco. How often do you go to each point-of-sale in the list below to buy cigarettes or roll-your-own tobacco?<br>• Supermarket<br>• Bar or restaurant<br>• Duty-free shop<br>• Via Internet<br>• Bookstore (for example Primera, AKO or Bruna)<br>• Tobacconist<br>• Gas station<br>• From someone else – not at a shop or other regular point of sale<br>1 Daily<br>2 Weekly<br>3 Monthly<br>4 Less than monthly<br>5 Never<br>99 Don't know/Don't want to say
Travel time to points-of-sale<br><br>[matrix question]	This question is about your living environment.<br>How many minutes on average does it take to get to the:<br>• Supermarket<br>• Bookstore(for example Primera, AKO or Bruna)<br>• Tobacconist<br>• Gas station<br>1 Less than 5 minutes<br>2 5-10 minutes<br>3 11-20 minutes<br>4 20-30 minutes<br>5 More than 30 minutes<br>9 Don't know/Don't want to say
Purchases abroad	How often in the last 6 months have you bought cigarettes or rolling tobacco from outside the Netherlands?

	1 Never<br>2 Only once<br>3 A few times<br>4 Many times<br>5 All of the time<br>9 Don't know/Don't want to say
Type of point-of-sale after most recent relapse	A relapse is defined as having a successful smoking quit attempt of at least 48 hours, followed by a return to continued tobacco smoking.<br>Where did you get cigarettes from after your most recent relapse?<br>1 Supermarket<br>2 Bar or restaurant<br>3 Duty-free shop<br>4 Via Internet<br>5 Bookstore (for example Primera, AKO or Bruna)<br>6 Tobacco shop<br>7 Gas station<br>9 From someone else – not at a shop or other regular point of sale<br>10 I already had cigarettes at home<br>99 Don't know/Don't want to say
Seeing tobacco in the retail environment	How often have you seen the following when visiting a store in the last month:<br>• Cigarette packages at the counter or checkout<br>• Smoking accessories (for example lighters, filters, rolling paper<br>• Advertisements for tobacco<br>• People smoking at the entrance of the store<br>• A friend buying cigarettes<br>• A family member buying cigarettes<br>1 Very often<br>2 Often<br>3 Sometimes<br>4 Rarely<br>5 Never<br>9 Don't know/Don't want to say
Illegal purchases	Have you bought cigarettes or tobacco during the past 6 months from people who sell cigarettes or tobacco independently, for example at the door or on the street?<br>1 Yes<br>2 No<br>9 Don't know/Don't want to say
	How many times in the past 6 months have you done this?<br>1 Once<br>2 A few times<br>3 Common<br>4 Always<br>9 Don't know/Don't want to say<br>10 Don't want to say
E-cigarette use	E-cigarettes are tobacco-free products that vaporise liquid. This can be a liquid with or without nicotine.<br>Have you ever used an e-cigarette?<br>1 Yes<br>2 No<br>3 I have never heard of e-cigarettes<br>9 Don't know/Don't want to say
Frequency of e-cigarette use	How often do you currently use an e-cigarette?<br>1 Daily<br>2 Weekly<br>3 Monthly<br>4 Not at all<br>9 Don't know/Don't want to say
Location of e-cigarette purchase – most often	Where do you usually buy (disposable) electronic cigarettes (including cartridges/liquid) for yourself?<br>1 Supermarket<br>2 Bar or restaurant<br>3 Duty-free shop<br>4 Via Internet<br>5 Bookstore (for example Primera, AKO or Bruna)<br>6 Tobacco shop<br>7 Vape shop<br>8 Gas station<br>9 From someone else – not at a shop or other regular point of sale<br>10 I don't buy it myself

	11 Other<br>99 Don’t know/Don’t want to say
Frequency of e-cigarette purchase	How often do you buy on average [disposable electronic cigarettes / electronic cigarette cartridges / electronic cigarette liquid]?<br>1 Almost every day<br>2 2-3 times a week<br>3 Once a week<br>4 2-3 times a month<br>5 Once a month<br>6 Less than once a month<br>9 Don’t know/Don’t want to say
Heated tobacco product (HTP) use	Electronic products that heat tobacco instead of burning it (so-called heated tobacco products or heat-not-burn) use batteries to heat capsules, sleeves, or sticks that look like cigarettes. These include products such as IQOS.<br>Have you ever used one of these heated tobacco products,<br>even one time?<br>1 Yes<br>2 No<br>3 I have never heard of heated tobacco products<br>9 Don’t know/Don’t want to say
Frequency of HTP use	How often do you currently use heated tobacco products?<br>1 Daily<br>2 Weekly<br>3 Monthly<br>4 Less than monthly<br>5 Not at all<br>9 Don’t know/Don’t want to say
Location of HTP purchase – most often	Where do you usually buy heated tobacco products for yourself? In other words, at which shop or point of sale?<br>1 Supermarket<br>2 Bar or restaurant<br>3 Duty-free shop<br>4 Via Internet<br>5 Bookstore (for example Primera, AKO or Bruna)<br>6 Tobacco shop<br>7 Vape shop<br>8 Gas station<br>9 From someone else – not at a shop or other regular point of sale<br>10 I don’t buy it myself<br>11 Other<br>99 Don’t know/Don’t want to say
Frequency HTP purchase	How often do you buy heated tobacco products on average?<br>1 Almost every day<br>2 2-3 times a week<br>3 Once a week<br>4 2-3 times a month<br>5 Once a month<br>6 Less than once a month<br>99 Don’t know/Don’t want to say
Support for supermarket ban	To what extent do you agree with the following statement? The sale of tobacco products in supermarkets should be completely banned.<br>1 Strongly agree<br>2 Somewhat agree<br>3 Neither agree nor disagree<br>4 Somewhat disagree<br>5 Strongly disagree<br>9 Don’t know/Don’t want to say
Effect of the supermarket ban – Reduce smoking [post-implementation of supermarket ban onwards]	We would now like to ask you a few questions about the effect that the ban of sale of cigarettes in supermarkets may have had on you:<br>To what extent has the sales ban made you cut down on the number of cigarettes you smoke?<br>1 Not at all<br>2 Just a little<br>3 A fair amount<br>4 A great deal<br>9 Don’t know/Don’t want to say
Effect of the supermarket ban – motivation to quit [post-implementation of supermarket ban onwards]	To what extent does the supermarket cigarette sales ban motivate you to stop smoking?<br>1 A great deal<br>2 A fair amount<br>3 Just a little

	4 Not at all<br>5 Don't know/Don't want to say
Undesired effect of supermarket ban – purchased greater volume of tobacco<br>[post-implementation of supermarket ban onwards]	Have you done any of the following because of the supermarket cigarette sales ban?<br><br>Purchased varieties that include more sticks or more tobacco per pack?<br>1 Yes<br>2 No<br>9 Don't know/Don't want to say
Undesired effect of supermarket ban – purchased greater quantities of tobacco<br>[post-implementation of supermarket ban onwards]	Purchased more packs of cigarettes or rolling tobacco per visit?<br>1 Yes<br>2 No<br>9 Don't know/Don't want to say
Impact supermarket ban on quitting/ staying abstinent<br>[post-implementation of supermarket ban onwards]	When you think about yourself, do you agree that the ban on selling cigarettes in supermarkets makes it easier for you to [quit smoking/stay abstinent]?<br>1 Strongly disagree<br>2 Disagree<br>3 Disagree/disagree<br>4 Agree<br>5 Totally agree<br>9 Don't know/Don't want to say
Attitude about quitting	If you quit smoking within the next 6 months, this would be…<br>1 Very wise<br>2 Wise<br>3 Neutral<br>4 Unwise<br>5 Very unwise<br>9 Don't know/Don't want to say
	If you quit smoking within the next 6 months, this would be…<br>1 Very pleasant<br>2 Pleasant<br>3 Neutral<br>4 Unpleasant<br>5 Very unpleasant<br>9 Don't know/Don't want to say
	If you quit smoking within the next 6 months, this would be…<br>1 Very positive<br>2 Positive<br>3 Neutral<br>4 Negative<br>5 Very negative<br>9 Don't know/Don't want to say
Self-efficacy for quitting	If you decided to give up smoking completely in the next 6 months, how sure are you that you would succeed?<br>1 Not at all sure<br>2 Slightly sure<br>3 Moderately sure<br>4 Very sure<br>5 Extremely sure<br>9 Don't know/Don't want to say
	How easy or hard would it be for you to quit smoking if you wanted to?<br>1 Not at all difficult<br>2 Slightly difficult<br>3 Moderately difficult<br>4 Very difficult<br>5 Extremely difficult<br>9 Don't know/Don't want to say
Control beliefs for quitters<br><br>[for quitters]<br>[Wave 2 onwards]	Will you be able to stay quit when…<br>• You have just woken up<br>• You have experienced something annoying<br>• You are having a cup of coffee or tea<br>• You are drinking alcohol<br>• You are offered a cigarette<br>1 Sure I could<br>2 Probably could<br>3 Maybe I could, maybe not<br>4 Probably could not<br>5 Sure I could not<br>9 Don't know/Don't want to say
Social norms	Thinking about the people who are important to you – how do you think most of them would feel about your quitting smoking within the next 6 months?

	1 Strongly disapprove<br>2 Disapprove<br>3 Neutral<br>4 Approve<br>5 Strongly approve<br>9 Don't know/Don't want to say<br><br><br>[version for quitters in post-measurement]<br>Thinking about the people who are important to you – how do you think most of them would feel about you staying abstinent?<br>1 Strongly disapprove<br>2 Disapprove<br>3 Neutral<br>4 Approve<br>5 Strongly approve<br>9 Don't know/Don't want to say
Social denormalisation	To what extend do you agree or disagree with the following statements?<br>People who are important to me believe I should not smoke.<br>1 Strongly agree<br>2 Agree<br>3 Not agree, but also not disagree<br>4 Disagree<br>5 Strongly disagree<br>9 Don't know/Don't want to say
	Society disapproves of smoking.<br>1 Strongly agree<br>2 Agree<br>3 Not even, but also not disagree<br>4 Disagree<br>5 Strongly disagree<br>9 Don't know/Don't want to say
	There are fewer and fewer places I [would] feel comfortable smoking.<br>1 Strongly agree<br>2 Agree<br>3 Not even, but also not disagree<br>4 Disagree<br>5 Strongly disagree<br>9 Don't know/Don't want to say
Seen or heard about smoking cessation campaign(s)	In the last 12 months about how often, if at all, have you seen or heard a smoking cessation campaign about smoking, such as the Smoke-free Generation?<br>1 Never<br>2 Rarely<br>3 Sometimes<br>4 Often<br>5 Very often<br>9 Don't know/Don't want to say
Seen or heard about smoking policy	In the last 12 months about how often, if at all, have you seen or heard of smoking policy in the news?<br>2 Rarely<br>3 Sometimes<br>4 Often<br>5 Very often<br>9 Don't know/Don't want to say
Triggers to think about quitting	To what extent have any of the following made you think about quitting smoking in the last 12 months?<br>• A prevention message or campaign<br>• The price of cigarettes<br>• The ban on sale of tobacco products in supermarkets<br>• Smoking bans in new or more places<br>• Availability of telephone helpline/ Quitline/ information line<br>1 A great deal<br>2 A fair amount<br>3 Just a little<br>4 Not at all<br>9 Don't know/Don't want to say
Age in years<br><br>[Received from Kantar panel data]	Age in years
Sex	Sex<br>1 Man

[Received from Kantar panel data]	2 Woman
Hoogst afgeronde opleiding<br><br>[Received from Kantar panel data]	What is the highest level of education you have completed?
Income	If you add up all the incomes within your household, what is the total gross monthly income? Gross income is the income before tax deductions and social security contributions and so on.<br>• Less than 750 € per month<br>• Between €750 and 1000<br>• Between €1000 and 1250<br>• Between €1250 and 1500<br>• Between €1500 and 1750<br>• Between €1750 and 2000<br>• Between €2000 and 2500<br>• Between €2500 and 3000<br>• Between €3000 and 4000<br>• Between €4000 and 5000<br>• Between €5000 and 6000<br>• Between €6000 and 7000<br>• €7000 and more<br>• Don't know/Don't want to say
Financial stress	In the last 30 days, because of a shortage of money, were you unable to pay any important bills on time, such as electricity, telephone or rent bills?<br>1 Yes<br>2 No<br>8 Don't know/Don't want to say
Perceived quality of neighbourhood	The following questions are about your neighbourhood. By neighbourhood, we mean the general area around your house where you might perform routine tasks, such as visiting with neighbours, going for a walk, shopping, or going to the park.<br>For the following questions, we would like you to think about your own neighbourhood when answering:<br><br>How satisfied are you with your neighbourhood?<br>1 Completely satisfied<br>2 Satisfied<br>3 Somewhat satisfied<br>4 Not very satisfied<br>5 Not at all satisfied<br>9 Don't know/Don't want to say
	How would you rate the quality of the air in your neighbourhood?<br>1 Very good<br>2 Good<br>3 Adequate<br>4 Poor<br>5 Very poor<br>9 Don't know/Don't want to say
	How would you rate the general upkeep of your neighbourhood?<br>1 Very good<br>2 Good<br>3 Adequate<br>4 Poor<br>5 Very poor<br>9 Don't know/Don't want to say
	To what extent are the following statements true:<br>This is a neighbourhood where I feel safe from personal attacks.<br>1 Very true<br>2 Mostly true<br>3 Somewhat true<br>4 Mostly untrue<br>5 Not at all true<br>9 Don't know/Don't want to say
	There are enough facilities and services to meet my needs in my neighbourhood. This could include recreational and sport facilities, health services and shops.<br>1 Very true<br>2 Mostly true<br>3 Somewhat true<br>4 Mostly untrue<br>5 Not at all true<br>9 Don't know/Don't want to say
	This is very little noise disturbance in my neighbourhood.<br>1 Very true<br>2 Mostly true

	3 Somewhat true<br>4 Mostly untrue<br>5 Not at all true<br>9 Don’t know/Don’t want to say
	SURVEY END

© 2023 Nagelhout G. E. et al.
